# A Clinical Decision Support Tool for Intimate Partner Violence Screening Among Women Veterans: Development and Qualitative Evaluation of Provider Perspectives

**DOI:** 10.2196/57633

**Published:** 2024-09-25

**Authors:** Fernanda S Rossi, Justina Wu, Christine Timko, Andrea L Nevedal, Shannon Wiltsey Stirman

**Affiliations:** 1 Department of Psychiatry and Behavioral Sciences Stanford University School of Medicine Stanford, CA United States; 2 Center for Innovation to Implementation (Ci2i) Veterans Affairs Palo Alto Health Care System Menlo Park, CA United States; 3 Center for Clinical Management Research Veterans Affairs Ann Arbor Health Care System Ann Arbor, CA United States; 4 National Center for PTSD Veterans Affairs Palo Alto Health Care System Menlo Park, CA United States

**Keywords:** intimate partner violence, clinical decision support, intimate partner violence screening, women veterans

## Abstract

**Background:**

Women veterans, compared to civilian women, are especially at risk of experiencing intimate partner violence (IPV), pointing to the critical need for IPV screening and intervention in the Veterans Health Administration (VHA). However, implementing paper-based IPV screening and intervention in the VHA has revealed substantial barriers, including health care providers’ inadequate IPV training, competing demands, time constraints, and discomfort addressing IPV and making decisions about the appropriate type or level of intervention.

**Objective:**

This study aimed to address IPV screening implementation barriers and hence developed and tested a novel IPV clinical decision support (CDS) tool for physicians in the Women’s Health Clinic (WHC), a primary care clinic within the Veterans Affairs Palo Alto Health Care System. This tool provides intelligent, evidence-based, step-by-step guidance on how to conduct IPV screening and intervention.

**Methods:**

Informed by existing CDS development frameworks, developing the IPV CDS tool prototype involved six steps: (1) identifying the scope of the tool, (2) identifying IPV screening and intervention content, (3) incorporating IPV-related VHA and clinic resources, (4) identifying the tool’s components, (5) designing the tool, and (6) conducting initial tool revisions. We obtained preliminary physician feedback on user experience and clinical utility of the CDS tool via the System Usability Scale (SUS) and semistructured interviews with 6 WHC physicians. SUS scores were examined using descriptive statistics. Interviews were analyzed using rapid qualitative analysis to extract actionable feedback to inform design updates and improvements.

**Results:**

This study includes a detailed description of the IPV CDS tool. Findings indicated that the tool was generally well received by physicians, who indicated good tool usability (SUS score: mean 77.5, SD 12.75). They found the tool clinically useful, needed in their practice, and feasible to implement in primary care. They emphasized that it increased their confidence in managing patients reporting IPV but expressed concerns regarding its length, workflow integration, flexibility, and specificity of information. Several physicians, for example, found the tool too time consuming when encountering patients at high risk; they suggested multiple uses of the tool (eg, an educational tool for less-experienced health care providers and a checklist for more-experienced health care providers) and including more detailed information (eg, a list of local shelters).

**Conclusions:**

Physician feedback on the IPV CDS tool is encouraging and will be used to improve the tool. This study offers an example of an IPV CDS tool that clinics can adapt to potentially enhance the quality and efficiency of their IPV screening and intervention process. Additional research is needed to determine the tool’s clinical utility in improving IPV screening and intervention rates and patient outcomes (eg, increased patient safety, reduced IPV risk, and increased referrals to mental health treatment).

## Introduction

### Background

Intimate partner violence (IPV), defined as physical or sexual violence; stalking; psychological, emotional, or verbal aggression; coercion; or economic abuse by an intimate partner [1], is a serious public health issue that disproportionately affects women. In the United States, >1 in 3 women have experienced IPV in their lifetime [2]. Compared to men, women are more likely to experience negative IPV-related outcomes, such as physical injury, fear, and posttraumatic stress disorder [1,3]. Women veterans, compared to civilian women, are especially at risk of experiencing IPV, with up to 60% of women veterans in relationships reporting experiencing IPV [4,5].

To address the increased IPV risk for women veterans and in line with current recommendations by the United States Preventative Services Task Force [6], in 2014 and updated in 2024, the Veterans Health Administration (VHA) issued a national directive recommending IPV screening and intervention across primary care and other VHA facilities nationwide [7]. As part of this rollout, the Women’s Health Clinic (WHC), a primary care clinic at the Veterans Affairs Palo Alto Health Care System (VAPAHCS), began piloting a paper-based IPV screening and intervention protocol, but preliminary evaluations revealed significant implementation barriers. Barriers included health care providers’ inadequate IPV training, competing demands, time constraints, discomfort addressing IPV, and making decisions about the appropriate type or level of intervention. These findings align with those of a larger qualitative study examining the implementation of IPV screening and intervention in women’s health primary care clinics across 11 VHA medical centers nationwide [8]. Such barriers are understandable given that health care providers screening for IPV are taxed with making complex decisions that require navigating varying risk levels associated with differing IPV interventions depending on the patient’s circumstances. Health care providers must weigh many factors, including patients’ mental and physical health, characteristics of the abuse and of the perpetrator, lethality risk due to IPV, and family and economic circumstances, to deliver care that minimizes the risk of danger for the patient.

Health care provider–related barriers regarding IPV screening and intervention may be addressed through the use of clinical decision support (CDS). CDS tools are computer-based systems that guide individuals through a decision-making process by providing intelligently filtered information at appropriate times in the clinical workflow for increasing health care quality and efficiency [9]. They can incorporate computerized alerts, clinical guidelines, patient-specific information and other contextual factors, documentation templates, and summary reports, among others [9]. CDS has been highly effective in the treatment of medical conditions, such as reducing cardiovascular risk in patients with type 2 diabetes [9], but the application of CDS to IPV screening and intervention is novel.

CDS, in the IPV context, can optimize the clinical decision-making process by efficiently guiding health care providers through IPV assessment, documentation, and intervention. Results from Kaiser Permanente, one of the first integrated health care systems to adopt a CDS approach to IPV screening and assess its impact on clinical care, have been promising [10]. They show that integration of a clinical reminder in the electronic health record to screen for IPV coupled with prompts on questions to ask as well as “smart links” to IPV materials (eg, safety planning tips and IPV community resources) was associated with a significant increase in mental health referrals [10]. However, more sophisticated and interactive IPV CDS tools that can offer health care providers intelligent step-by-step guidance on how to conduct IPV screening and intervention according to patient factors have yet to be developed. For example, existing tools fail to differentiate between women reporting higher levels of IPV (eg, severe physical violence) who may need extensive resources and a safety plan or lower levels of IPV (eg, only yelling and calling names) who may need different and less intensive resources. A more advanced step-by-step IPV CDS tool can enhance the quality of IPV screening and intervention by being sensitive to patient factors and facilitate the decision-making process for health care providers.

### Objectives

This study sought to describe (1) the development of a novel, interactive, step-by-step IPV CDS tool prototype for physicians in the VAPAHCS WHC and (2) physicians’ preliminary feedback on user experience and clinical utility of the CDS tool. This study provides an example of the process used to develop an interactive, step-by-step IPV CDS tool and how such a tool may be used to address IPV screening and intervention implementation barriers in a primary care clinic. Interactive, step-by-step IPV CDS has yet to be applied in the VHA and other health care systems more broadly and holds considerable promise for ensuring efficient implementation of best IPV care practices and, consequently, improving the value, accessibility, and quality of health care delivered to women veterans and civilians. In turn, this can lead to improved IPV-related patient outcomes, such as increased use of mental health treatment and reduced IPV risk.

## Methods

### Overview

The study’s process for developing and testing the IPV CDS tool was based on the CDS model development process by Coulter et al [11]. This process describes the main elements or general stages involved in systematically developing a CDS tool, including determining the tool’s scope and design, developing a prototype, testing the prototype with relevant individuals in an iterative process, testing the prototype under real-world conditions, and final development of the tool [11]. This study focuses only on the initial 3 stages (ie, determining the tool’s scope and design, developing a prototype, and testing the prototype). Steps 1 to 4 of phase 1, described in the following paragraphs, fall within the scope and design stage. Steps 5 and 6 of phase 1 fall within the prototype development stage. Phase 2 falls within the prototype testing stage.

### Phase 1: Develop the IPV CDS Tool Prototype

#### Step 1: Identify the Scope of the IPV CDS Tool

The first author (FSR), during a 6-month clinical psychology rotation at the VAPAHCS WHC, directly and systematically observed physicians’ challenges with IPV screening and intervention, participated in discussions about these challenges at monthly meetings and daily morning rounds, and individually interviewed 4 physicians about their experiences addressing IPV. Observations and informal evaluations resulting from these activities were captured through informal written notes and highlighted barriers to providing IPV care at the VAPAHCS WHC, including physician discomfort addressing IPV and making decisions about the appropriate type or level of intervention. We used the information gathered to determine the tool’s goal (to develop a step-by-step, interactive IPV CDS tool), target users (VAPAHCS WHC physicians), and scope (IPV screening and intervention).

#### Step 2: Identify IPV Screening and Intervention Content

The IPV screening and intervention content within the tool was informed by various sources: (1) a literature review that identified current evidence-informed IPV care practices and clinical recommendations, specifically literature related to IPV screening (eg, who, when, and how to screen) and interventions (eg, types of interventions and how to communicate with an individual experiencing IPV), focusing on brief interventions that may be conducted by health care providers in primary care. We specifically examined the literature for recommendations on which interventions may be most effective and appropriate according to IPV type (eg, physical violence and psychological aggression), level (eg, high vs low), and risk of harm (eg, high vs low)—allowing us to design the tool to reveal appropriate interventions based on the patients’ IPV screening results. (2) For areas in which there was not a clear recommendation given a lack of evidence, we consulted with IPV experts, including health care workers (ie, clinical psychologists, women’s health primary care providers, and social workers) with IPV expertise, IPV researchers, and Veterans Affairs IPV Assistance Program (IPVAP) coordinators (ie, staff dedicated to assisting with VHA IPV screening and prevention services). (3) We also relied on the study team members’ extensive IPV expertise, including the first author’s (FSR) IPV-focused research and clinical experience as a licensed clinical psychologist. (4) In addition, we reviewed the VHA’s guidelines, developed by the IPVAP, for conducting IPV screening and intervention.

**Figure 1:**
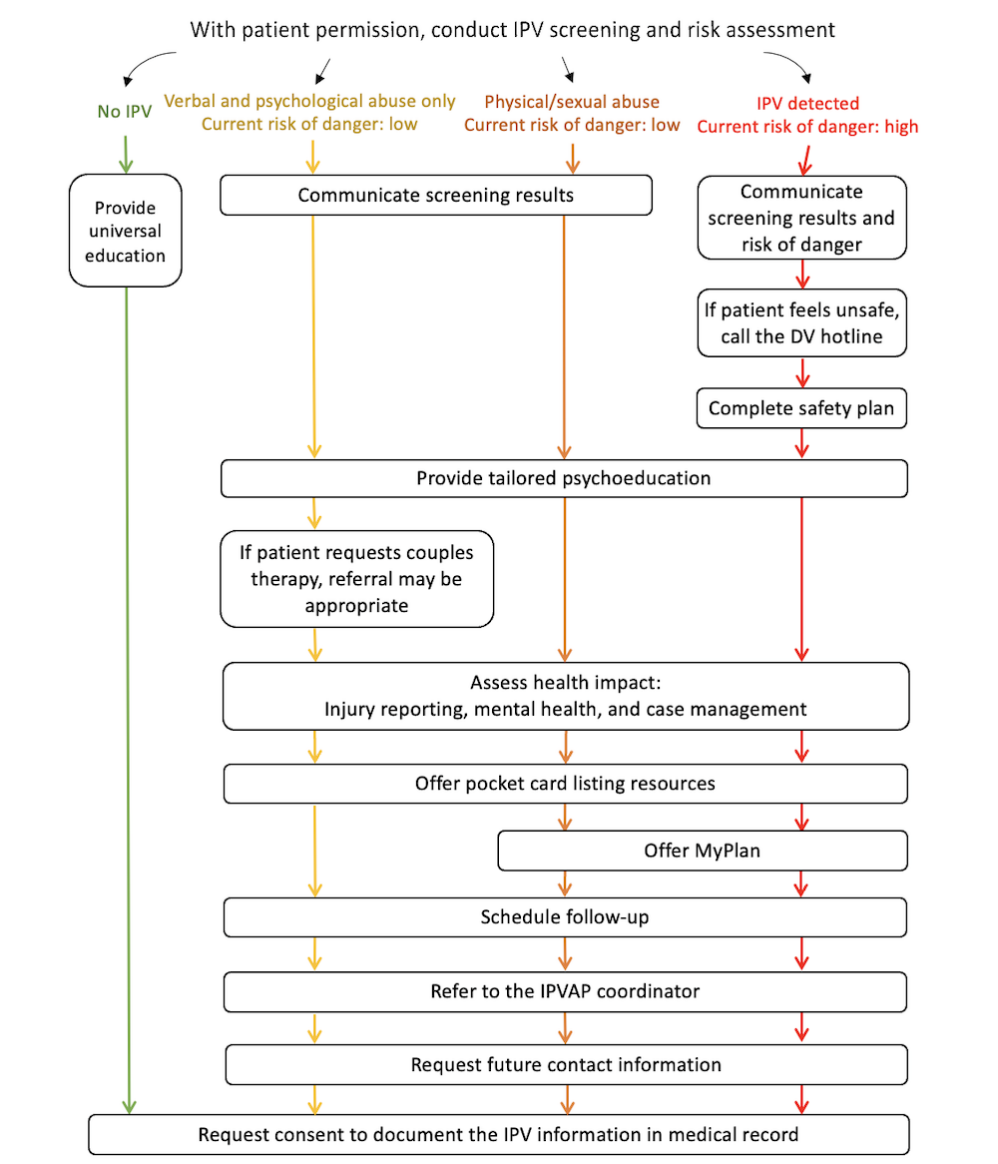
The decision tree embedded within the intimate partner violence (IPV) clinical decision support tool for women’s health primary care providers to conduct IPV screening and intervention with women veterans. DV: domestic violence; IPVAP: IPV Assistance Program.

On the basis of these sources, we developed a decision tree (Figure 1) connecting differing IPV screening results to appropriate brief IPV interventions, such as safety planning (eg, MyPlan, a web-based safety planning tool [12]), providing IPV resources (eg, a domestic violence hotline number), making appropriate referrals to ongoing support services (eg, mental health counseling and social services), scheduling a follow-up appointment, and providing IPV- or relationship-focused psychoeducation.

We focus the IPV CDS tool on brief IPV interventions for the following reasons: (1) There is insufficient direct evidence that screening for IPV alone, without any response or intervention, can reduce IPV-related harms [6]. (2) Brief interventions, compared to longer-term, intensive IPV interventions, are more feasible in primary care given time constraints. (3) Access to longer-term, intensive interventions shown to help with IPV, such as mental health treatment or other support services, can be limited immediately after IPV screening. Thus, brief interventions (eg, providing resources and referrals) may help link patients to such services. (4) Some brief interventions show empirical support in addressing IPV. For example, MyPlan, an interactive web-based tool that guides individuals experiencing IPV in creating a safety plan, has been shown to increase women’s use of IPV-related safety behaviors [12]. (5) The brief interventions selected for this IPV CDS tool are in line with VHA guidelines (eg, providing resources and referrals and safety planning) [7]. Thus, the IPV CDS tool developed in this study helps address health care provider barriers in being able to implement the VHA’s existing guidelines.

The brief interventions selected for the tool are all existing interventions that have been empirically tested [6,12-14] or recommended in guidelines. Some of the selected brief interventions are appropriate for addressing any reported IPV risk or experience (eg, providing IPV resources), while other brief interventions may be appropriate for addressing greater IPV risk (eg, safety planning). Figure 1 and phase 1 results provide a more in-depth description of the differing IPV screening results categorizations within the IPV CDS tool and the recommended brief interventions for each category.

When selecting the brief interventions, we also considered their potential harms when delivered incorrectly. Although there is inadequate evidence to determine the harms of IPV brief interventions [6], certain brief interventions when delivered incorrectly may theoretically be harmful to individuals experiencing IPV. For example, it is contraindicated to place full responsibility on the individual experiencing IPV to create a safety plan and establish safety mechanisms without any guidance. Given this consideration, we designed the IPV CDS tool to recommend safety planning in conjunction with a professional (ie, a health care provider and domestic violence hotline specialist) when the risk of danger is high or under the guidance of MyPlan [12] if the risk of danger is low.

#### Step 3: Incorporate IPV-Related VHA and Clinic Resources

We collected IPV-related VHA and VAPAHCS WHC clinic resources that would allow us to tailor the IPV CDS tool to the VAPAHCS WHC setting (eg, IPV patient brochures already used by the clinic, lists of phone numbers for local police stations, documents to complete mandated state reporting for IPV, older-adult abuse, and child abuse). We also incorporated the VHA’s nationally recommended IPV screening tools (eg, use of the Extended-Hurt, Insult, Threaten, Scream [E-HITS] IPV screening tool [15] and use of a subset of items from the Danger Assessment [16] to assess risk of harm) and materials (eg, safety plan template for IPV experience).

#### Step 4: Identify CDS Components

We used the framework for CDS systems [17] to identify core components of the IPV CDS tool and determine its format, structure, and features. This framework outlines 24 axes as part of the CDS workflow, each of which we considered for potential relevance (a list of the 24 axes is provided in Multimedia Appendix 1). For example, we determined how to deliver IPV-related information to health care providers (eg, on the web), the reasoning method used to present information (eg, built-in rule-based algorithms), the extent to which health care providers can interact with the tool (eg, pop-down menus and fill in the blanks), and the extent to which the tool can be customized for each patient (eg, the tool shows safety planning information for patients reporting higher levels of IPV but not for those reporting lower levels of IPV and no safety concerns).

#### Step 5: Tool Design

Using information collected in steps 1 to 4, we designed the prototype, using Qualtrics, a web-based survey platform [18]. Future work will focus on adapting the IPV CDS tool to the electronic medical record system and using artificial intelligence to enhance the tool’s decision-making process.

#### Step 6: Initial Revisions

After developing the IPV CDS tool prototype, we obtained initial informal feedback from IPV experts (ie, 2 health care workers with IPV expertise, 3 IPV researchers, and 1 IPVAP coordinator). Specifically, the first author (FSR) held 30- to 60-minute videoconferencing meetings with each expert and demonstrated the tool through screen sharing while allowing the expert to freely share their thoughts and feedback on the tool. The first author took notes during the meetings, capturing their feedback. The IPV CDS tool was revised according to this initial feedback, at which point the tool was ready for preliminary testing.

### Phase 2: Obtain Preliminary Physician Feedback on the IPV CDS Tool

#### Recruitment

Consistent with purposeful criterion sampling [19], the sampling pool consisted of all primary care physicians involved in IPV screening at the VAPAHCS WHC, a primary care clinic that specializes in women veterans’ health. The VAPAHCS WHC health care providers have specialized training in gender-specific (eg, women’s wellness exam) and non–gender-specific care (eg, diabetes) from the woman veteran perspective. We identified the sampling pool with help from the VAPAHCS WHC director. Recruitment involved emailing physicians an invitation for study participation. We emailed physicians a study invitation 2 additional times, each 1 week apart, in the case of nonresponse. A total of 5 (45%) of the 11 invited physicians declined to participate or did not respond to recruitment efforts. We recruited 6 VAPAHCS WHC primary care physicians, which was sufficient for reaching data saturation. Data saturation was demonstrated when physicians in the final interviews (eg, fourth, fifth, and sixth interviews) reported similar feedback to physicians in the initial interviews on key questions regarding the tool’s design, layout, content, features, and functionality. Thus, no additional recruitment efforts were needed. Physicians who agreed to be interviewed scheduled a virtual interview and received a meeting link. Shortly before the scheduled meeting, physicians were emailed a link to the IPV CDS tool.

#### Approach

Physicians participated in a 30- to 60-minute interview using videoconferencing software and a semistructured interview guide based on the Health IT Usability Evaluation Model [20]. Interviews were conducted by author JW, a female researcher with a master’s degree in public health and expertise in qualitative and health services research. Physicians had no relationship with or knowledge of the interviewer before the interview. The interview asked physicians to engage in different tasks (eg, complete a safety plan using the tool) and answer questions related to usability and implementation (example interview questions are provided in Multimedia Appendix 2). Following the interview, physicians were asked to complete the System Usability Scale (SUS) [21], a 10-item standardized measure for assessing usability (eg, I felt very confident using the tool—1 meaning *strongly disagree* to 5 meaning *strongly agree*). SUS scores range from 0 to 100, with scores >68 demonstrating above-average usability. Interviews were audio-recorded and transcribed verbatim by the VHA’s Centralized Transcription Services Program.

#### Statistical Analysis

Reporting of qualitative findings adheres to the COREQ (Consolidated Criteria for Reporting Qualitative Research) checklist (Multimedia Appendix 3) [22]. Interview data were analyzed using rapid qualitative analysis [23,24] to extract actionable feedback to inform design updates and improvements. First, 2 authors (FSR and JW) used a template to summarize each transcript and field notes taken during the interview. Summaries were used to identify patterns and illustrative quotes according to the following deductively derived domains: design and layout (appearance), information and content (specificity, amount, and quality of information), features and functionality (technical capability), real-world application (use of the tool in everyday practice), implementation (use of the tool in own practice and clinic), and overall impressions. The authors then met to review their summaries of physician feedback across domains and resolve discrepancies. Next, the authors used an Excel (Microsoft Corp) spreadsheet to create a matrix from these summaries, displaying subject domains and the corresponding responses and quotes from each participant. The matrix was used to create summaries of each domain that included recurring patterns, specific recommendations, and quotes.

### Ethical Considerations

This quality improvement project was determined to be exempt from institutional review board review. Physicians did not receive compensation for their participation in the qualitative interviews, as it is against federal policy to provide additional compensation to Department of Veterans Affairs employees. Despite a determination of nonresearch, physicians gave their informed consent to participate in the interview and audio record the conversation. Physicians were informed that their information would be kept confidential and recordings would be transcribed with identifiable information removed. We requested that physicians not state their name or any other identifiable information during the interview.

## Results

### Phase 1: The IPV CDS Tool

The IPV CDS prototype is a computerized tool that WHC physicians could access with the appropriate link. The tool has two main sections: (1) assessment and (2) intervention. The assessment section includes a validated IPV screening tool (ie, the E-HITS per VHA guidelines) with follow-up questions from the Danger Assessment [16], inquiring about the risk of lethality due to IPV (also per VHA guidelines) to be administered to each patient during the health encounter. Information provided within the assessment section determines the information displayed in the intervention section by use of a decision tree. Figure 1 shows an illustration of the decision tree embedded within the IPV CDS tool. Overall, depending on the information provided within the assessment section, patients are categorized as having a negative or positive IPV screen. For patients who have a negative IPV screen, the IPV CDS tool recommends that physicians provide universal education regarding healthy relationships, as is recommended in the literature [25]. The tool offers a script and links to handouts that physicians can use to engage patients in this discussion.

Patients who have a positive IPV screen are categorized into one of three categories based on the patient’s reports of IPV in the past year and the risk assessment: (1) verbal or psychological abuse only and low current risk of danger due to IPV (ie, only verbal or psychological abuse items on the E-HITS were endorsed, and none of the 5 risk items were endorsed), (2) physical or sexual violence and low current risk of danger due to IPV (ie, at least 1 of the physical or sexual violence items on the E-HITS was endorsed, and none of the risk items were endorsed), and (3) any type of IPV and high current risk of danger due to IPV (ie, at least 1 item on the E-HITS was endorsed, and at least 1 of the risk items was endorsed). Before displaying information regarding the appropriate intervention, the tool shows summaries of the IPV assessment and the recommended intervention. The IPV CDS tool, then, displays separate screens for each step in the intervention process. These steps differ according to the IPV and risk category. For example, for patients in category 1, the tool recommends that physicians, with the patient’s permission, provide tailored IPV psychoeducation, assess the health impact of IPV, hand the patient a pocket card with local IPV-related resources, schedule a follow-up appointment, and refer the patient to the IPVAP coordinator. In addition, physicians are advised to ask for methods of safe contact and consent to document IPV-related information in the electronic health record. Finally, the tool displays a tailored summary of the assessment results and intervention components that the physician can copy and paste into their clinical note. For patients in category 2, the tool recommends an additional step for physicians to inform patients of a web-based safety planning tool (MyPlan) [12]. For patients in category 3, the tool recommends a second additional step by instructing physicians on how to complete a safety plan with the patient or assisting the patient in calling the domestic violence hotline to complete the safety plan. Across all categories, the tool provides links to important documents (eg, safety plan templates and state reporting forms) and tailored example scripts incorporating trauma-sensitive language that physicians can use to communicate with patients. For instance, each category includes an example script to help physicians communicate screening results and deliver tailored psychoeducation appropriate for that category. The category 1 script, for example, is tailored to include information about verbal or psychological abuse (eg, “Half of women experience the behaviors you reported, which we call psychological/verbal abuse”). This information is expanded to include other forms of IPV in the category 2 and 3 scripts (eg, “About 1 of every 3 women report experiencing IPV”). The example scripts are short to help physicians deliver information efficiently yet effectively. To improve physician efficiency, we included a note on the psychoeducation screen allowing physicians to skip this step if they are short on time.

### Phase 2: Physician Feedback on the IPV CDS Tool

#### Overview

All participants interviewed were female and practicing women’s health physicians. Of the 5 physicians who provided demographic information, 3 (60%) self-identified as Asian and 2 (40%) as White. Similarly, 3 (60%) physicians were aged between 30 and 39 years, and 2 (40%) were aged between 60 and 69 years. A total of 4 (80%) physicians indicated ≥5 years of medical experience. The mean SUS score across physicians was 77.5 (SD 12.75), suggesting good tool usability.

Next, we describe physician feedback on 6 core domains for assessing the preliminary tool. Textbox 1 shows sample quotations related to each domain.

**Qualitative feedback from Veterans Affairs Palo Alto Health Care System—Women’s Health Clinic primary care providers on the intimate partner violence clinical decision support tool.**

**Design and layout**

- User-friendly
  “I think the design is really user-friendly. Like, it’s very easy to kind of go through each of the screens, and the response to each question follows in a very methodical manner. There’s no part of it where I’m like—Oh, what’s going on? Why am I on this screen?...The amount of information on each page is very reasonable, too.” [Physician 002]
- Sequence of steps
  “...because you can’t give a safety plan without actually knowing what’s going on physically with the patient, because the plan will then depend upon what you find.” [Physician 005]
- Length
  “If there was a way to pare this down to less screens. I think it’s really, really good information.” [Physician 002]
  “You know those PowerPoints, you begin with a bullet or two, people digest that, then you click down on other additional information. So, it’s not too much information right up front.” [Physician 005]

**Information and content**

- Level of detail
  “This is really nice for continuing knowledge of physicians to know that these are the forms and these are the phone numbers, so that when, just between colleagues, as these cases come up especially in the VA [Veterans Affairs], they can kind of informally disseminate that knowledge. And it’s good learning for the colleagues, for this to be embedded in their heads, since they’re going to be doing it at every wellness visit.” [Physician 002]
- Need for additional information
  “...we could populate our own shelters in here, because I will guarantee you, primary care doesn’t know where those shelters are.” [Physician 003]

**Features and functionality**

- Strengths and weaknesses of features
  “...it’s nice that you have the ticker at the top that kind of lets providers know how far into the process they are.” [Physician 002]
  “I think filling the tool along the way is best. Like, anything to decrease the amount of documentation we have to do after the fact, I think is best. And if it happens as we go on, like that’s the most efficient way.” [Physician 001]

**Real-world application**

- Need for tool
  “If somebody uses the tool and [a patient] screens positive, especially with the high-risk questions, it’s basically going to be very, very time consuming to do this follow up, and it probably will be the bulk of the visit. That is not a bad thing, because if somebody is having that level of IPV [intimate partner violence], I don’t really want to talk to them about their blood pressure. I want to talk to them about this.” [Physician 006]
  “As a screening tool, I think it’s a bit lengthy and a bit wordy, especially if you’re thinking of somebody who doesn’t have any exposure to this, maybe only has learned about IPV once or twice in medical school and maybe once or twice in residency. But I think it’s really, really good for training, for providers to sit down and be able to read through this in a relaxed setting.” [Physician 002]

**Implementation**

- Feasibility
  “This takes over the visit and it can put a little wrench in your schedule, but it’s no different from kind of the other times that that happens in primary care...And a tool like this is really great, because it lays it all out for you.” [Physician 002]
  “I think the physicians will be using a lot of it. I think a lot of us really buy in to screening, but don’t always feel super comfortable with the next steps, if somebody discloses, so I think this would be super helpful.” [Physician 006]
  “[Physicians who are not primary care providers] would want to know what information is in the tool, so that it aligns with how they talk to the patient as well, but they will not be doing the screening themselves.” [Physician 001]
- Competing demands
  “There’s a lot of screening for all kinds of primary care things...And it’s really hard to address all of those things in one appointment [especially] if it’s for some more acute thing.” [Physician 001]

**Overall impression**

- “I think it would make [my confidence] go up, for sure, in a much more concrete way. I think I had ideas before about what I can do in the clinic, but this tool gives me concrete steps of like calling the hotline, and some of the things that I would have to search for online that I wouldn’t be able to find quickly, are all in this tool.” [Physician 001]

#### Design and Layout

##### User-Friendly

Physicians expressed that the tool was user-friendly due to its predictable presentation and the reasonable amount of information on each screen.

##### Sequence of Steps

Some physicians thought the order in which steps were presented was logical, but others made recommendations about how to improve the order of steps; for example, injury reporting should come before safety planning.

##### Length

Physicians indicated that the tool had many steps, which would take significant time to complete. The solutions suggested were including a navigation menu that lets steps be skipped, simplifying and reducing the number of steps, and including ways to opt out of steps. Another suggestion was to enable parts of the content to be hidden and expanded.

#### Information and Content

##### Level of Detail

Physicians liked the tool’s detailed information (eg, example scripts for communicating risk of harm to patients, phone numbers and links to safety plans, and injury reporting forms) and thought it could serve as a resource where information is stored and organized. Physicians thought the tool would be particularly helpful for physicians who are less experienced with IPV care or may facilitate disseminating IPV-related information to other physicians.

##### Need for Additional Information

Physicians requested adding additional information, such as more details about injury, police, and child abuse reporting. Physicians requested more tailored information that would facilitate the screening and intervention process (eg, a list of domestic violence shelters in the area).

#### Features and Functionality

Physicians liked various features of the tool, such as the ticker at the top that indicates how much of the process they have completed or the ability to copy and paste patient disposition from the tool into a note. However, participants also indicated features to strengthen the tool, such as populating the tool along the way to decrease documentation at the end or having a menu to access specific information.

#### Real-World Application

Physicians liked the IPV CDS tool and found it clinically useful because it could help address anxiety related to not knowing how to appropriately conduct IPV screening and intervention. Physicians indicated that the IPV CDS tool would help them provide IPV care, especially physicians less familiar with IPV screening and intervention. Some suggested it could serve as a training tool for new physicians. Physicians particularly found the example scripts in the tool helpful for discussing IPV with patients. When a patient is seeking help for a non–IPV-related issue, physicians thought the tool could be too time consuming and leave little time to address other health care problems; however, physicians felt it was important to prioritize IPV.

#### Implementation

##### Feasibility

Overall, physicians thought implementation of the IPV CDS tool was feasible because it provides all the necessary information to complete IPV screening and intervention. One recommendation to ensure successful implementation was to appoint a champion to teach staff about the tool.

Physicians stated that primary care providers, social workers, and psychologists would all want the IPV CDS tool, though each would use it differently, and some may not need all aspects of the tool. Most thought the tool should be implemented in primary care, while ensuring that other nonprimary care providers are aware of the information provided in the tool.

##### Competing Demands

Most physicians cited the time required to complete the tool as the primary barrier to its implementation. Physicians have to complete screenings for multiple conditions, so agenda setting and prioritizing can become a challenge.

#### Overall Impression

Physicians liked the IPV CDS tool, found it helpful for addressing patients reporting IPV, and thought it increased their confidence in conducting IPV screening and intervention. The main drawback was the length of the tool, and physicians recommended streamlining the content to accommodate the time constraints of clinic visits.

## Discussion

### Principal Findings

This study sought to develop a novel and interactive, step-by-step CDS tool for IPV screening and intervention to be implemented in a VHA women’s primary care clinic. Findings indicate that the IPV CDS tool was clinically useful and generally well received by physicians. Physicians found the tool helpful and needed in their practice and stated that it would increase their confidence in managing patients reporting IPV. Physicians thought implementation of the tool in primary care was feasible. When such IPV CDS tools are successfully adopted, they may increase the quality of IPV screening and intervention in primary care and, in turn, improve IPV-related outcomes (eg, increase use of mental health treatment and reduce IPV experience), though this warrants further research.

Findings also highlighted several important enhancements that must be considered in future tool revisions or when developing similar IPV CDS tools. For instance, one issue is designing an IPV CDS tool that is comprehensive and informative yet sufficiently concise to fit the clinic workflow. When designing the IPV CDS tool used in this study, we incorporated IPV care practices recommended by the literature, experts, and VHA guidelines. Still, many physicians indicated that the tool, when encountering patients with high risk, was too time consuming, leaving little time to address any other needs. Though some physicians thought the tool was time consuming, they also recognized the importance of dedicating the health encounter to an issue as important as IPV. Nonetheless, when CDS tools disrupt the clinic workflow, physicians may be less likely to use them or more likely to override the tool recommendations, diminishing the tool’s clinical value [26]. While it is critical to ensure that information displayed within the tool is concise, removing or skipping critical screening or intervention practices may negatively impact the well-being and safety of the patient. Therefore, it is essential, when designing IPV CDS tools, to find solutions that meet the needs of physicians but also do not compromise patient health. Solutions include (1) keeping only the tool components that are most critical to patient safety (eg, using a standardized IPV screening measure and evidence-based interventions) and (2) conducting rigorous and iterative testing of the tool, improving the tool’s efficiency (eg, more concise language and easier tool navigation) until it meets the demands of physicians, patients, and the clinic [26]. This helps maximize the tool’s adoption by ensuring that it is feasible, acceptable to both physicians and patients, and can be well integrated into the clinic workflow.

An equally important issue is designing an IPV CDS tool that is flexible (ie, there is flexibility within the tool and with how the tool is used). More flexible CDS tools can give physicians a greater sense of autonomy in their practice, rather than feeling directed, which in turn promotes higher adoption of the tool [27]. When considering flexibility within the tool, it is critical to determine features or functions that help physicians flexibly navigate the tool and have easy access to important information. For instance, physicians in this study requested a navigational menu for easier use of the IPV CDS tool.

When considering flexibility with how the tool is used, it is critical to determine the intended purposes of the tool. For example, physicians in this study questioned whether the IPV CDS tool could be used for additional purposes, including as an educational tool for less experienced physicians and a checklist for more experienced physicians. IPV CDS tools that serve multiple purposes may result in greater uptake and impact but may be more challenging to design, as they can include more sophisticated technological features and must meet the demands of differing target audiences and contexts. One solution is to consult with physicians before designing the tool to determine which features and uses of the tool may be most helpful in improving IPV care.

Even when IPV CDS tools are flexible and fit the clinic workflow, it is important that they be specific. Physicians in this study indicated wanting more specific and tailored information in the tool (eg, a list of local shelters). While including this information can help facilitate and increase efficiency of the IPV care process, IPV CDS tools that have highly specific and tailored information may not be generalizable to other settings and may need frequent updates when information changes. To help address this, it may be necessary to conduct regular monitoring of the tool to ensure that information is still accurate [26].

The aforementioned physician concerns will be used to inform the next iteration and testing of the IPV CDS tool. IPV CDS tools that address these concerns have greater adoption potential and, therefore, increased capacity to positively impact IPV care. Despite room for improvement, our findings suggest an important need for our IPV CDS tool to improve the quality of IPV care.

### Limitations

This study had several limitations, which point to areas of future research. Our findings are based on only women’s health physicians and 1 clinic. While this clinic provided us with an ideal setting from which to obtain feedback from physicians, future studies should gather data from other settings and differing health care providers who also conduct IPV care. Future usability evaluations of the tool could also gather additional physician feedback on tool features and content that were not a direct focus of this study, such as the psychoeducation content. As our focus was solely on the usability of the IPV CDS tool, further evaluation is needed to assess its impact on clinical outcomes, such as increased IPV screening by physicians and reduced IPV experience among patients. We did not interview patients; thus, research is also needed examining patient perspectives on the acceptability of the IPV CDS tool.

Furthermore, this study does not offer a solution to situations where an abusive partner refrains from leaving the patient alone in a health encounter, thus preventing physicians from completing IPV screening and intervention. Asking about IPV in front of an abusive partner may result in underreporting of IPV or, if reported, can endanger the patient’s life due to potential partner retaliation. To protect the patient, the IPV screening and intervention steps outlined in the IPV CDS tool need to be completed privately and confidentially. This is a significant limitation in the IPV screening and intervention process given that these patients, who are likely experiencing severe IPV, will not have access to appropriate IPV care. We recommend that clinic staff discuss and implement creative solutions to help address this problem. For example, the VAPAHCS WHC displays informational IPV posters in the women’s restroom stalls. In addition, there are stacks of pocket cards with IPV resources in the women’s restroom for women veterans to take with them. It may also be possible to develop an IPV CDS tool that uses artificial intelligence to screen for IPV using behavioral and verbal observations. If IPV is detected in these cases, physicians would need to discreetly intervene, such as providing IPV resources through a general health brochure or a follow-up phone call.

### Implications for Practice and Policy

This study offers an example of how other clinics and health care systems can adapt the current interactive, step-by-step IPV CDS tool to potentially help primary care providers conduct IPV screening and intervention. While this study focuses on primary care due to the VHA’s recommendations for IPV screening in primary care, it is important to consider other health care settings where health care providers may benefit from an IPV CDS tool, such as health care providers in mental health specialty clinics. The IPV CDS tool will likely need to be tailored to each setting depending on health care providers’ needs. For example, health care providers in mental health specialty clinics may have time during the health encounter to conduct more in-depth IPV screening and extensive interventions. This study also highlights various challenges that may emerge when creating interactive, step-by-step IPV CDS tools regardless of the setting and offers solutions to address these challenges. Findings demonstrate that interactive, step-by-step CDS tools for IPV screening and intervention are promising in addressing some physician barriers to IPV screening and intervention. If adopted, such tools have the potential to transform IPV screening and intervention in health care settings by standardizing the process, increasing the frequency at which screening is performed, and guaranteeing that patients experiencing IPV are receiving the recommended care to improve outcomes. However, additional research is needed to further determine their clinical utility in improving IPV screening and intervention rates and patient outcomes (eg, increased patient safety, reduced IPV risk, and increased referrals to mental health treatment).

### Conclusions

We developed a novel clinical decision tool to assist women’s health care providers with IPV screening and intervention. Initial user experiences are encouraging. Widespread adoption of similar decision tools may help streamline IPV screening and intervention processes and increase use of best practices. When screening or best practices are ignored, even by well-intentioned health care providers, patients experiencing IPV are put at increased risk of harm.

## Appendix

Multimedia Appendix 1 The 24 axes in the framework for classifying decision support systems.

Multimedia Appendix 2 Example interview questions for obtaining provider feedback on the clinical decision support tool.

Multimedia Appendix 3 COREQ (Consolidated Criteria for Reporting Qualitative Research) checklist.

## Abbreviations

CDS: clinical decision support
COREQ: Consolidated Criteria for Reporting Qualitative Research
E-HITS: Extended-Hurt, Insult, Threaten, Scream
IPV: intimate partner violence
IPVAP: Intimate Partner Violence Assistance Program
SUS: System Usability Scale
VAPAHCS: Veterans Affairs Palo Alto Health Care System
VHA: Veterans Health Administration
WHC: Women’s Health Clinic

## Data Availability

The data sets generated and analyzed during this study are available from the corresponding author on reasonable request.

## References

[1] Intimate partner violence. Center for Disease Control and Prevention.

[2] Black MC, Basile KC, Breiding MJ, Smith SG, Walters ML, Merrick MT, Chen J, Stevens MR (2011). The National Intimate Partner and Sexual Violence Survey: 2010 summary report. National Center for Injury Prevention and Control, Centers for Disease Control and Prevention.

[3] Caldwell JE, Swan SC, Woodbrown VD (2012). Gender differences in intimate partner violence outcomes. Psychol Violence.

[4] Dichter ME, Cerulli C, Bossarte RM (2011). Intimate partner violence victimization among women veterans and associated heart health risks. Womens Health Issues.

[5] Iverson KM, Vogt D, Maskin RM, Smith BN (2017). Intimate partner violence victimization and associated implications for health and functioning among male and female post-9/11 veterans. Med Care.

[6] Curry SJ, Krist AH, Owens DK, Barry MJ, Caughey AB, Davidson KW, Doubeni CA, Epling JW, Grossman DC, Kemper AR, Kubik M, Kurth A, Landefeld CS, Mangione CM, Silverstein M, Simon MA, Tseng CW, Wong JB, US Preventive Services Task Force (2018). Screening for intimate partner violence, elder abuse, and abuse of vulnerable adults: US preventive services task force final recommendation statement. JAMA.

[7] (2024). VHA directive 1198: intimate partner violence assistance program. Department of Veterans Affairs.

[8] Iverson KM, Adjognon O, Grillo AR, Dichter ME, Gutner CA, Hamilton AB, Stirman SW, Gerber MR (2019). Intimate partner violence screening programs in the veterans health administration: informing scale-up of successful practices. J Gen Intern Med.

[9] Tcheng JE, Bakkan S, Bates DW, Lomotan EA, Mackay E, Bonner H, Gandhi TK, Teich J, Josephs M, Weingarten S (2017). Optimizing strategies for clinical decision support: summary of a meeting series. National Academy of Medicine.

[10] McCaw B (2011). Using a systems-model approach to improving IPV services in a large health care organization. Institute of Medicine.

[11] Coulter A, Stilwell D, Kryworuchko J, Mullen PD, Ng CJ, van der Weijden T (2013). A systematic development process for patient decision aids. BMC Med Inform Decis Mak.

[12] Glass NE, Perrin NA, Hanson GC, Bloom TL, Messing JT, Clough AS, Campbell JC, Gielen AC, Case J, Eden KB (2017). The longitudinal impact of an internet safety decision aid for abused women. Am J Prev Med.

[13] Rhoades GK, Stanley SM (2011). Using individual-oriented relationship education to prevent family violence. J Couple Relatsh Ther.

[14] Sabri B, Tharmarajah S, Njie-Carr VP, Messing JT, Loerzel E, Arscott J, Campbell JC (2022). Safety planning with marginalized survivors of intimate partner violence: challenges of conducting safety planning intervention research with marginalized women. Trauma Violence Abuse.

[15] Iverson KM, King MW, Gerber MR, Resick PA, Kimerling R, Street AE, Vogt D (2015). Accuracy of an intimate partner violence screening tool for female VHA patients: a replication and extension. J Trauma Stress.

[16] Campbell JC, Webster DW, Glass N (2009). The danger assessment: validation of a lethality risk assessment instrument for intimate partner femicide. J Interpers Violence.

[17] Sim I, Berlin A (2003). A framework for classifying decision support systems. AMIA Annu Symp Proc.

[18] (2005). Home page. Qualtrics.

[19] Palinkas LA, Horwitz SM, Green CA, Wisdom JP, Duan N, Hoagwood K (2015). Purposeful sampling for qualitative data collection and analysis in mixed method implementation research. Adm Policy Ment Health.

[20] Brown W, Yen PY, Rojas M, Schnall R (2013). Assessment of the health IT usability evaluation model (Health-ITUEM) for evaluating mobile health (mHealth) technology. J Biomed Inform.

[21] Brooke J (1986). SUS - a quick and dirty usability scale. Redhatch Consulting Ltd.

[22] Tong A, Sainsbury P, Craig J (2007). Consolidated criteria for reporting qualitative research (COREQ): a 32-item checklist for interviews and focus groups. Int J Qual Health Care.

[23] Gale RC, Wu J, Erhardt T, Bounthavong M, Reardon CM, Damschroder LJ, Midboe AM (2019). Comparison of rapid vs in-depth qualitative analytic methods from a process evaluation of academic detailing in the Veterans Health Administration. Implement Sci.

[24] Nevedal AL, Reardon CM, Opra Widerquist MA, Jackson GL, Cutrona SL, White BS, Damschroder LJ (2021). Rapid versus traditional qualitative analysis using the Consolidated Framework for Implementation Research (CFIR). Implement Sci.

[25] Miller E, McCaw B (2019). Intimate partner violence. N Engl J Med.

[26] Sutton RT, Pincock D, Baumgart DC, Sadowski DC, Fedorak RN, Kroeker KI (2020). An overview of clinical decision support systems: benefits, risks, and strategies for success. NPJ Digit Med.

[27] Ford E, Edelman N, Somers L, Shrewsbury D, Lopez Levy M, van Marwijk H, Curcin V, Porat T (2021). Barriers and facilitators to the adoption of electronic clinical decision support systems: a qualitative interview study with UK general practitioners. BMC Med Inform Decis Mak.

