# Supplementary material for: A Clinical Decision Support Tool for Intimate Partner Violence Screening Among Women Veterans: Development and Qualitative Evaluation of Provider Perspectives
Source: JMIR Form Res. 2024 Sep 25;8:e57633. doi: 10.2196/57633 (PMC11464933; doi:10.2196/57633)
Supplement: Multimedia Appendix 1 [file formative_v8i1e57633_app1.docx]

| Category | Axes |
| --- | --- |
| **Context** | Clinical setting |
|  | Clinical task |
|  | Unit of optimization |
|  | Relation to point of care |
|  | Potential external barriers to action |
| **Knowledge/**  **Data Source** | Clinical knowledge source |
|  | Data source |
|  | Data source intermediary |
|  | Data coding |
|  | Degree of customization |
|  | Update mechanism |
| **Decision Support** | Reasoning method |
|  | Clinical urgency |
|  | Recommendation explicitness |
|  | Logistical complexity of recommended action |
|  | Response requirement |
| **Information Delivery** | Delivery format |
|  | Delivery mode |
|  | Action integration |
|  | Delivery interactivity/explanation availability |
| **Workflow** | System user |
|  | Target decision maker |
|  | Output intermediary |
|  | Degree of workflow integration |
