# Supplementary material for: A Clinical Decision Support Tool for Intimate Partner Violence Screening Among Women Veterans: Development and Qualitative Evaluation of Provider Perspectives
Source: JMIR Form Res. 2024 Sep 25;8:e57633. doi: 10.2196/57633 (PMC11464933; doi:10.2196/57633)
Supplement: Multimedia Appendix 2 [file formative_v8i1e57633_app2.docx]

Background

1. Do you regularly screen patients for intimate partner violence? If so,
   1. What is your current practice for screening patients for intimate partner violence?
   2. How many patients would you say you’ve screened in the past month?
2. On a scale of 1 to 5, where 1 is not confident at all and 5 is very confident, how confident do you feel at this time in your ability to screen for intimate partner violence and appropriately intervene if there is a positive screen?

Task 1: Tool click-through

1. What are your first thoughts about this tool?
2. What do you think of the design and layout? Probes if needed:
   1. What are your thoughts on being able to find what you’re looking for? Being able to get around in the tool?
   2. What do you think about the amount of information provided?

Task 2: Review recommendations screen

1. What are your thoughts about the information on this page?
2. Based on this screen, what do you do next?
3. Is there anything you’d change about this screen?

Task 3: Make a Safety Plan

1. How confident do you feel about being able to complete a safety plan with your patient?
   1. Are there any barriers to being able to complete the safety plan?
2. What could be added or changed that would make this screen more helpful to you?

Task 4: Review talking points for communicating risk of harm

1. How helpful are these talking points in communicating risk of IPV-related harm to patients? Probes if needed:
   1. What could be added or changed to help you communicate risk of IPV-related harm?

Task 5: Review summary screen

1. How useful is this information?
2. What could be added or changed?

Implementation

1. Now that you have a sense of what the tool does and how to use it, do you think you would be able to use this tool in your women’s health practice? Why or why not?
2. How do you think the intimate partner violence screening tool will impact the workflow in your setting?
3. In your setting, who do you think will be most likely to want this tool implemented? Who will be most likely to use it?
4. What level of support is there for improving intimate partner violence screening from clinic and/or facility leaders in your setting?
5. What kinds of high-priority initiatives or activities are already happening in your setting that may impact implementation of the tool?
6. At the beginning of the interview, you indicated that your confidence level on a scale of 1 to 5 regarding your ability to screen for intimate partner violence and appropriately intervene in the case of a positive screen is a ___. Do you believe that using this tool would change your confidence level? How would you rate your confidence level now, on a scale of 1 to 5?
